# Sexual Interaction in Digital Contexts and Its Implications for Sexual Health: A Conceptual Analysis

**DOI:** 10.3389/fpsyg.2021.769732

**Published:** 2021-11-30

**Authors:** Nicola Döring, Nicole Krämer, Veronika Mikhailova, Matthias Brand, Tillmann H. C. Krüger, Gerhard Vowe

**Affiliations:** ^1^Media Psychology and Media Design, Institute of Media and Communication Science, Department of Economic Sciences and Media, Technische Universität Ilmenau, Ilmenau, Germany; ^2^Social Psychology: Media and Communication, Department of Computer Science and Applied Cognitive Science, Faculty of Engineering, University of Duisburg-Essen, Duisburg, Germany; ^3^General Psychology: Cognition and Center for Behavioral Addiction Research (CeBAR), Department of Computer Science and Applied Cognitive Science, Faculty of Engineering, University of Duisburg-Essen, Duisburg, Germany; ^4^Department of Psychiatry, Social Psychiatry and Psychotherapy, Section of Clinical Psychology and Sexual Medicine, Hannover Medical School, Hannover, Germany; ^5^Communication and Media Studies, Center for Advanced Internet Studies (CAIS), Bochum, Germany

**Keywords:** internet sexuality, cybersex, online sexual activities (OSA), sexting, pornography, sex robots, sexual consent, commercial sex

## Abstract

Based on its prevalence, there is an urgent need to better understand the mechanisms, opportunities and risks of *sexual interaction in digital contexts* (SIDC) that are related with sexual arousal. While there is a growing body of literature on SIDC, there is also a lack of conceptual clarity and classification. Therefore, based on a conceptual analysis, we propose to distinguish between sexual interaction (1) *through*, (2) *via*, and (3) *with* digital technologies. (1) Sexual interactions *through* digital technologies are face-to-face sexual interactions that (a) have been started digitally (e.g., people initiating face-to-face sexual encounters through adult dating apps) or (b) are accompanied by digital technology (e.g., couples augmenting their face-to-face sexual encounters through filming themselves during the act and publishing the amateur pornography online). (2) Sexual interactions *via* digital technology are technology-mediated interpersonal sexual interactions (e.g., *via* text chat: cybersex; *via* smartphone: sexting; *via* webcam: webcam sex/camming). (3) Sexual interactions *with* digital technology occur when the technology itself has the role of an interaction partner (e.g., sexual interaction with a sex robot or with a media persona in pornography). The three types of SIDC and their respective subtypes are explained and backed up with empirical studies that are grouped according to two major mediators: consent and commerce. Regarding the causes and consequences of the three types of SIDC we suggest a classification that entails biological, psychological, social, economic, and technological factors. Regarding implications of SIDC we suggest to focus on both opportunities and risks for sexual health. The proposed conceptual framework of SIDC is meant to inform future research.

## Introduction

A significant amount of digital media use is sexuality-related and involves, for example, online pornography, sex dating apps, webcam sex, or the use of remote-controlled sex toys. Studies show that 68% of digital media users have been involved in some sort of sexual interaction in digital contexts (Döring and Mohseni, 2018). There is a growing body of literature on sexuality-related Internet and smartphone use and its effects, particularly its public health and sexual health impacts (e.g., sexual addiction and aggression, but also sexual pleasure, intimacy, and well-being). The literature database PubMed alone documents 5127 publications for the search term combination “online” and “sexual^∗^” in the paper title or abstract (as of August 2021). However, there is still a lack of conceptual clarity and classification of different types of sexuality-related activities that involve digital technologies. Hence, the aim of this conceptual analysis article is to develop a conceptual framework that covers and structures different types of sexual interaction in digital contexts and helps to disentangle opportunities and risks for sexual health.

## Definition of Sexual Interaction in Digital Contexts

The literature offers several umbrella terms to address sexuality-related digital media use such as “Internet sexuality” or “online sexual activities” (see Table 1). One problem with some of these concepts is, that they focus on the Internet as the key technology. As the digital media landscape is ever changing and more people are involved in sexuality-related activities with their smartphones (Anzani et al., 2018), there is a need to complement the old umbrella terms “Internet sexuality” and “online sexual activities” with new terms such as “smartphone sexuality” and “mobile sexual activities”, respectively. As possibly more people will be involved in sexuality-related activities with social and sexual robots in the future (Döring et al., 2020), one would need an additional term such as “robot sexuality” or “robot-enabled sexual activities” to address this emerging field. Avoiding a surplus of technology-specific terms, we suggest to refer to “digital contexts” of sexuality-related activities instead, to be as technology-inclusive as possible.

**TABLE 1:** Umbrella terms for sexuality-related digital technology use and their conceptual breadth.

**Term**	**Definition**	**Face-to-face interaction**	**Technology-mediated interaction**	**Human-technology interaction**	**Technology inclusiveness**	**Arousal focus**
Internet sexuality	Sexuality-related content and activities on the Internet (Döring, 2009).	+	+	+	–	–
Online sexual activities (OSA)	The use of the Internet for any type of activity that involves human sexuality (Cooper et al., 2001).	+	+	+	–	–
Digisexuality	Sexual experiences that are enabled or accompanied by digital technology (McArthur and Twist, 2017).	+	+	+	+	–
Cyberintimacy	Technology-mediated communication between existing and potential sexual partners (Kwok and Wescott, 2020).	–	+	–	+	–
Cybersex	A subcategory of OSA, a real-time online sexual interaction between two or more people (Daneback et al., 2005).	–	+	–	–	+
Sexting	The use of mobile devices or computers to send or receive sexually explicit messages, photographs, or images (Klettke et al., 2014).	–	+	–	–	+
Technology mediated sexual interaction (TMSI)	Interpersonal interaction with the use of digital technology that includes self-created sexually explicit content (Courtice and Shaughnessy, 2017).	–	+	–	+	+
Sexual interaction in digital contexts (SIDC)	Interaction associated with sexual arousal that involves the use of digital media content or a digital artifact, or takes place in an online or digital environment.	+	+	+	+	+

*Aspects that are covered within each umbrella term are marked with “+”. Respectively, aspects that are not addressed within selected umbrella term are marked with “−“.*

The umbrella term “digisexuality” (see Table 1) has already been introduced and attempts to overcome a too narrow technology focus as it includes a variety of digital technologies (McArthur and Twist, 2017). However, this umbrella term covers both sexuality-related activities that are related to sexual arousal (e.g., using online pornography, exchanging sexual messages with a steady partner or with a stranger) but also sexuality-related activities that are not related to sexual arousal (e.g., searching for sexual health information, campaigning for sexual rights with an online activist group). Both the causes and the consequences of arousal-oriented and non-arousal-oriented activities differ substantially (e.g., people might develop an addictive use of arousal-oriented but not of non-arousal-oriented applications; Griffiths, 2012). Hence, we suggest not to mix arousal and non-arousal activities.

Looking for umbrella terms that specifically focus on arousal-related sexual activities in digital contexts, we identified the terms “cyberintimacy”, “cybersex”, “sexting”, and “technology-mediated sexual interaction”. However, at a closer look, they were either not sufficiently arousal-oriented (e.g., “cyberintimacy”) or not sufficiently technology-inclusive (e.g., “cybersex”, “sexting”). That leaves “technology-mediated sexual interaction” as the seemingly best term (see Table 1).

The problem with the concept “technology-mediated sexual interaction” (TMSI), though, is its focus on one type of sexual interaction only, namely the interpersonal interaction that is mediated by technology (e.g., a couple living in a long-distance relationship experiencing sexual intimacy with each other *via* an online videoconference system; Courtice and Shaughnessy, 2017). When people deal with digital technologies in an arousal-oriented sexual manner, two further types of interaction are relevant: The technology can not only mediate the interpersonal interaction, it can also enable and shape a sexual face-to-face interaction (e.g., an online dating app enables offline sexual encounters among people who would not have met without the app; Timmermans and Courtois, 2018). Furthermore, there is considerable sexual interaction between the user and the technology itself in the role of an interaction partner, particularly with AI (artificial intelligence)-enhanced technologies such as software sexbots and hardware sex robots (e.g., people engaging in sexual interactions with a sex robot; Szczuka and Krämer, 2017b). These two additional and relevant types of interaction are not covered by the TMSI concept.

Hence, we propose the concept S*exual Interaction in Digital Contexts* (SIDC) as a new umbrella term that is technology-inclusive, arousal-oriented and covers three types of sexual interactions (see Table 1). We define SIDC as interaction associated with sexual arousal that involves the use of digital technology. According to the *Media Equation Approach* and the *Computers Are Social Actors* (CASA) *Approach*, it is theoretically and empirically well established that people experience and treat media content and digital technologies like social actors (Reeves and Nass, 1996; Krämer, 2008), thus the term “interaction” is applicable to sexual interactions between people, between people and digital media content (e.g., between a person and a media persona such as a porn actor on the screen), and between people and digital artifacts in both virtual and material form (e.g., between a person and a software sexbot or a hardware sex robot). The idea that people can in fact be involved in meaningful social and sexual interactions with media personas or with anthropomorphic artifacts is also a core element of the theory of *Para-Social Interactions* (PSI) and *Para-Social Relationships* (PSR; Horton and Wohl, 1956; Dibble et al., 2016).

Sexual interaction in digital contexts covers arousal-oriented interactions that are either *solitary-arousal* activities (e.g., digital pornography use during masturbation) or *partnered-arousal* activities (e.g., digital pornography use during sexual intercourse; Shaughnessy et al., 2017). The term SIDC does not include sexuality-related non-arousal activities in digital environments such as sexuality-related online information search, sexual health communication, or political online activism by and for sexual minorities.

## Conceptual Analysis of Sexual Interaction in Digital Contexts

This conceptual analysis article explores the concept of SIDC. A “concept” is understood as an abstraction of a phenomenon that is defined by its components and their interrelations (Jabareen, 2009). To identify and structure the main components of SIDC, we underwent an analytical process adopted from related process models suggested in the literature on conceptual analysis (Bröder et al., 2019 drawing on Jabareen, 2009 and Rodgers, 2000) that entailed six main steps:

1. Searching for relevant theoretical and empirical English-language contributions on the target concept of SIDC (sexual interaction in digital contexts).
2. Reading and categorizing the selected publications.
3. Identifying and naming the main dimensions and components of the target concept based on the literature.
4. Deconstructing the dimension and component attributes, (re)grouping and integrating the dimensions and components into one conceptual model through several rounds of brainstorming and discussion among authors.
5. Validating and revising the conceptual model through critical discussions within the academic setting.
6. Identifying hypotheses and implications for future research and development regarding the target concept based on the conceptual model.

### Three Types of Sexual Interaction in Digital Contexts

At the core of the resulting conceptual framework of SIDC is the differentiation of the three already mentioned types of interaction: sexual interaction *through*, *via* and *with* digital technology (see Table 2). Each of the three types has several subtypes that will be elaborated below.

**TABLE 2:** Classification and illustration of the three main types of SIDC: through, via, with.

**Type of SIDC**	**Definition**	**Example activities**
Sexual interaction ***through*** digital technologies	Interpersonal sexual interactions in face-to-face contexts that have been started digitally or are accompanied by digital technology.	People initiating their face-to-face sexual interactions *through* adult dating apps. People shaping their face-to-face sexual interactions *though* live filming and streaming the act on the Internet.
Sexual interaction ***via*** digital technologies	Interpersonal sexual interactions that take place in computer-mediated contexts or within an interactive immersive virtual reality system.	People experiencing a computer-mediated interpersonal sexual interaction *via* sexually explicit text-based, photo-based, audio-based or video-based communication with each other. People experiencing a computer-mediated interpersonal sexual interaction *via* an immersive virtual reality system.
Sexual interaction ***with*** digital technologies	Sexual interactions between the person and the technological artifact in the role of an interaction partner.	People interacting *with* 2D and 3D Internet pornography and their respective sexual media personas. People interaction *with* physical technological artifacts such as AI-enhanced sex chatbots, sex dolls, or sex robots.

### Five Types of Causes and Consequences of Sexual Interaction in Digital Contexts

Further core components of the SIDC model are the causes and consequences of the different types of sexual interactions through, via, and with technology. The conceptual analysis led to a five-component model of causes and consequences that cover biological, psychological, social, economic and technological factors (see Table 3). This conceptualization is based on the *Bio-Psycho-Social Model of Health* (Engel, 1977; Lindau et al., 2003) and the *Bio-Psycho-Social-Model of Sexuality* (Shaeer et al., 2017; Leavitt et al., 2021). The bio-psycho-social model encourages to look at health and sexuality not as something purely biomedical or physical, but to acknowledge the multiple biological, psychological and social dimensions involved. To the well-established bio-psycho-social model of health and of sexuality with thousands of publications we added economic and technological factors as both are particularly important in sexual interaction in digital contexts: Access to some digital contexts (e.g., digital dating services such as Tinder or Grindr) requires economic resources, while active-productive participation in some digital contexts (e.g., adult content platforms such as PornHub or MyDirtyHobby) provides economic resources. This conceptualization of five types of causes and consequences of SIDC integrates a large variety of variables discussed in the literature in a structured way (see Table 3). For each type of sexual interaction in digital contexts (through, via, with) multiple biological, psychological, social, economic and technological causes and consequences are expected.

**TABLE 3:** Classification and illustration of the five types of causes and consequences of SIDC.

**Causes of SIDC**	**Example causes**	**Consequences of SIDC**	**Example consequences**
Biological causes	High level of testosterone and related increased sexual desire push user to sexuality-related technology use (e.g., exploration of sex dating apps).	Biological consequences	Use of sex dating apps enables face-to-face encounters with multiple sexual partners inaccessible before, leading to increased risk of STI (sexually transmitted infections).
Psychological causes	High level of anxiety due to relationship trauma makes user turn to a seemingly safer artificial sex partner (e.g., sex robot).	Psychological consequences	Use of a sex robot provides safe and satisfying experiences leading to stress reduction, sexual satisfaction, increased emotional stability.
Social causes	A sexual partner urges the user to sexual interaction in digital contexts (e.g., engage in unwanted sexting with them).	Social consequences	Experiences of unwanted and non-consensual sexual interactions in digital contexts cause, among other things, social withdrawal and relationship problems.
Economic causes	Lack of money pushes user to commercial sexual activities in digital contexts (e.g., commercial camsex).	Economic consequences	Providing services on a commercial camsex platform secures a living.
Technological causes	Technological affordances such as easy access and perceived anonymity pull the user to becoming a member of an online platform (e.g., online pornography platform).	Technological consequences	Use of an online pornography platform motivates user to try out additional technologies (e.g., better headphones, remote controlled sex toys linked to 3D pornography).

In public and academic discourses on SIDC negative general health outcomes (e.g., depression) and negative sexual health outcomes (e.g., addictive sexual behaviors, sexual aggression, sexual victimization, HIV/STI transmission) are a major concern (Döring, 2009). Often it has been assumed, that sexual expression in digital contexts is unnatural, risky and harmful per se (Rimm, 1994; Bull and McFarlane, 2000; Dwyer, 2005; Diliberto and Mattey, 2009). However, it has also been argued, that digital contexts offer new opportunities for helpful and beneficial sexual exploration (Dir and Cyders, 2015). Particularly the *Positive Sexuality Approach* (Williams et al., 2015) and the *Positive Technology Approach* (Riva et al., 2012), both rooted in the *Positive Psychology Approach* (Seligman and Csikszentmihalyi, 2014), are applicable to SIDC and stress the multiple opportunities for sexual and overall well-being. From the perspective of the digital technology users themselves, but also external observers such as researchers and clinical experts, some SIDC consequences are clearly evaluated as negative health outcomes (e.g., increased sexual frustration, insecurity, anxiety, trauma), while others as positive outcomes (e.g., improved sexual well-being, intimacy, confidence, pleasure).

Empirical research shows that people from the general population involved in SIDC tend to report both negative and positive outcomes, with positive outcomes often predominating (Shaughnessy et al., 2014; Döring and Mohseni, 2018; Courtice et al., 2021). However, type and intensity of reported individual consequences and overall outcomes vary greatly depending on the selection of outcome measures, the characteristics of the digital technology users (e.g., age, gender), and the type of sexual interaction involved (e.g., Lefkowitz and Vasilenko, 2014; O’Sullivan, 2014).

### Two Mediators of Sexual Interaction in Digital Contexts

Our conceptual analysis led to the identification of two main mediators: consent and commerce.

#### Consent: (Non)consensual Sexual Interaction in Digital Contexts

The literature has recognized many forms of non-consensual sexual interactions in digital contexts based on peer pressure, extortion, deception, threat etc. (Henry and Powell, 2018). The causes, characteristics and consequences of non-consensual sexual interactions differ significantly from consensual interactions. For example, consensual sexting is linked with positive consequences (e.g., pleasure, trust, confidence), while non-consensual sexting is linked with negative consequences (e.g., fear, anger, depression, self-harm; Wachs et al., 2021). Hence, consent is integrated as a mediator or intervening variable in our SIDC model.

The digital media user can be in the role of both the victim of non-consensual activities of others (e.g., being urged to participate in unwanted sexing) or the perpetrator (e.g., urging somebody else to participate in unwanted sexting; van Ouytsel et al., 2021). The literature describes consensual as well as non-consensual sexual interactions through, via, and with technology (e.g., Zytko et al., 2021). Examples of non-consensual interactions are sexual harassment or rape in face-to-face interactions enabled through sex dating apps (e.g., as revealed in the ABC News documentary “Tinder: Investigation reveals the dark side of the dating app”; ABC News In-depth, 2020), unwanted initiation of sexual interactions via technology (e.g., webcam exhibitionism in front of children or non-consenting adults; Jones, 2010), and violent interactions with technological artifacts (e.g., so-called “rape” of a sex robot; Danaher, 2017).

#### Commerce: (Non)commercial Sexual Interaction in Digital Contexts

Commerce is the second mediator integrated in the model. By definition, commercial sexual interactions involve the exchange of money or its equivalent, while non-commercial sexual interactions do not (Harcourt and Donovan, 2005). The relevance of commercial sex in digital contexts is based on both a growing supply and a growing demand in this field (Bernstein, 2007; Cunningham and Kendall, 2011; Minichiello et al., 2013; Sanders et al., 2018): People formerly not involved in commercial sex have now started both providing and buying commercial sexual services in digital contexts because digital contexts seem to be safer and more discreet than offline contexts. The literature addresses both non-commercial sex and commercial sex in digital contexts, each characterized by different causes, characteristics and consequences (Hakim, 2015; Jones, 2015).

The digital technology user can be in the role of both the commercial service provider (e.g., providing a live sex show via webcam for paying subscribers; Bleakley, 2014) or the client (e.g., paying to watch a live sex show via webcam; Weiss, 2018). While some authors describe low-threshold commercial webcam sex as a problem because it expands the commercial sex market and fosters a general commodification of sex, other authors point to the advantages of mediated commercial sex free from any risk of STI transmission or physical violence. Further examples of commercial SIDC are the use of digital technologies by sex workers to search for new offline clients (e.g., advertising own services in specialized apps or on personal websites; Pajnik, 2015) and digital artifacts being involved in sex work (e.g., “sex robot brothels” where one can rent a sex robot; Döring et al., 2020). Some authors expect positive social consequences of sex robots when they substitute commercial sex workers while they expect negative consequences of sex robots when they substitute non-commercial sexual partners (e.g., Yeoman and Mars, 2012; Woodward, 2020).

### Causal Interrelations Between Elements of the Sexual Interaction in Digital Contexts Model

The interrelations between the different dimensions and components are another core element of any conceptual model (Jabareen, 2009; Bröder et al., 2019). As SIDC addresses arousal-oriented sexual media and technology use behavior, we appropriated the most inclusive media use and effects theory available in the literature, the *Differential Susceptibility to Media Effects Model* (DSMM; Valkenburg and Peter, 2013), that has already been successfully applied to sexuality-related digital media use (Peter and Valkenburg, 2016).

**FIGURE 1:**
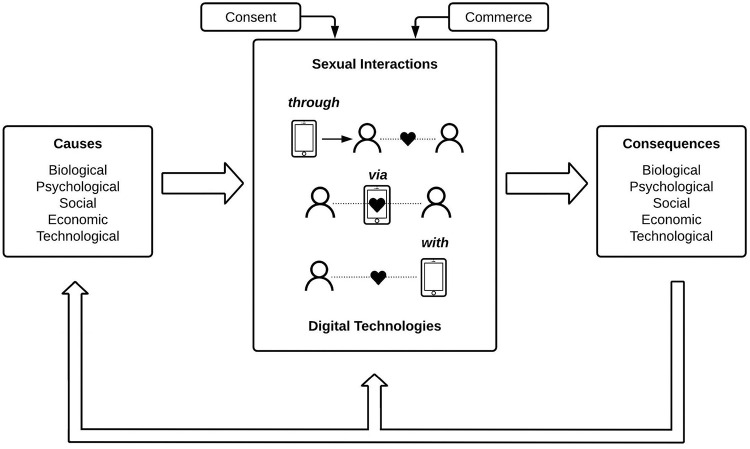
Conceptual model of sexual interaction in digital contexts (SIDC).

The model rejects media deterministic assumptions that simply explain media effects with media characteristics. Instead, it emphasizes the importance of individual predispositions for media use. These predispositions not only influence as predictor variables which digital media technologies are used in what ways; they also influence as moderator variables which immediate reactions to media use arise, which are precursors of media effects. Finally, the DSMM conceptualizes media effects as transactional factors that in turn influence predispositions, media use and the reactions experienced after media use. These repercussions of the media effects on future media use and media experiences are covered in Figure 1. There are also interdependencies between the causes (e.g., biological factors such as sexual hormone status can interact with psychological factors such as sexual motivation) and between the consequences (e.g., a social consequence such as a relationship breakup due to cyberinfidelity is often linked with psychological and economic consequences as well). Those interdependencies are not depicted in Figure 1, though, for the sake of clarity of the visualization.

### The Sexual Interaction in Digital Contexts Model

The final SIDC model including the three types of sexual interaction through, via, and with digital technology, the five types of causes and consequences, the two mediators consent and commerce and the main causal interrelations of the components is visualized in Figure 1.

The model will be elaborated further in the following sections that address each type of sexual interaction (through, via and with digital technology) separately with its respective subtypes.

## Sexual Interaction *Through* Digital Technologies

Sexual interaction *through* digital technologies is a SIDC type that focuses on the face-to-face sexual encounter and how it is enabled or shaped through digital technology use.

### Three Subtypes

For sexual interaction *through* digital technologies three subtypes can be identified (see Table 4).

**TABLE 4:** Sexual interactions THROUGH digital technologies: subtypes.

**Subtype**	**Activity**	**Consent**	**Commerce**	**Example studies**
(1) Face-to-face sexual interactions enabled through digital technology	People initiating their face-to-face sexual encounters *through* digital dating services.	+	–	Jung et al. (2019) Sevcikova and Daneback (2011) Timmermans and Courtois (2018) Wu and Ward (2018)
		–	–	Greene-Colozzi et al. (2020) Malesky (2007) Schulz et al. (2016) Thompson (2018)
		+	+	Brennan (2017) Kingston and Smith (2020) Mergenthaler and Yasseri (2021) Morris (2021)
		–	+	Beckham and Prohaska (2012) Jonsson et al. (2014) O’Brien and Li (2020)
(2) Face-to-face sexual interactions accompanied by passive-receptive use of digital technology	People shaping their face-to-face sexual encounters *through* joint pornography use.	+	–	Kohut et al. (2018) Willoughby and Leonhardt (2020)
		–	–	Langevin and Curnoe (2004)
		+	+	/
		–	+	/
(3) Face-to-face sexual interactions accompanied by active-productive use of digital technology	People shaping their face-to-face sexual encounters *through* live filming and streaming the act.	+	–	Ruberg (2016) Schwarz (2010)
		–	–	Eaton et al. (2020)
		+	+	Hofer (2014) Stardust (2019)
		–	+	Boyle (2011)

*Consent: “ + ” – consensual sexual interactions, “–“ – non-consensual sexual interactions. Commerce: “ + ” – commercial sexual interactions, “–“ – non-commercial sexual interactions.*

#### Using Digital Media to Search for Face-to-Face Sexual Interactions

The first subtype addresses people initiating face-to-face sexual interactions *through* social media platforms and digital dating services (see Table 4). According to the main mediators consent and commerce, the literature describes this subtype very differently: consensual non-commercial initiation of offline sexual encounters in digital contexts is often characterized as an interesting (albeit somewhat risky) opportunity to improve one’s social and sexual life (Sevcikova and Daneback, 2011; Hobbs et al., 2017; Timmermans and Courtois, 2018; Wu and Ward, 2018; Jung et al., 2019). Attempts of non-consensual initiation of offline sexual encounters in digital contexts are described, however, as unethical and illegal grooming of children or harassment of adults (Malesky, 2007; Thompson, 2018; Greene-Colozzi et al., 2020). When it comes to commercial sex, the literature acknowledges opportunities of reaching clients on social media or dating platform for self-determined sex workers of different genders and sexual identities (Brennan, 2017; Kingston and Smith, 2020; Mergenthaler and Yasseri, 2021; Morris, 2021). At the same time, digital technologies are also characterized as dangerous because young women in particular can be lured and pressured by older men into the digital paysex market without them being able to fully consent, sex trafficking takes place online and prostitutes are contacted online by offenders (Beckham and Prohaska, 2012; Jonsson et al., 2014; O’Brien and Li, 2020; see Table 4).

#### Using Digital Media Content During Face-to-Face Sexual Interactions

The second subtype focuses on people shaping their face-to-face sexual encounters *through* joint use of sexually explicit digital media content (see Table 4). Watching digital pornography together directly before and/or during sexual activities can shape the face-to-face sexual encounter in such a way that the content triggers couples to speak more openly about their sexual needs, inspires them to try out new sexual practices or enhances and prolongs their arousal (Sun et al., 2016; Kohut et al., 2018; Johnson et al., 2019; Willoughby and Leonhardt, 2020). This consensual use pattern needs to be differentiated from non-consensual use where the victim is forced to participate in joint pornography watching. For example, offenders against children sometimes force their victims to watch pornography together with them (Langevin and Curnoe, 2004). While consensual and non-consensual use of pornography may play a role in commercial sexual encounters as well, we could not find research on these issues (see Table 4).

#### Producing Digital Media Content During Face-to-Face Sexual Interactions

The third subtype addresses people shaping their face-to-face sexual encounters *through* joint recording, streaming or otherwise digitally documenting their sexual endeavors (see Table 4). This activity is often referred to as the production of amateur pornography (Ruberg, 2016). Some individuals and couples digitally document and share their sexual activities just out of curiosity and fun (Schwarz, 2010), while others do it with commercial interest in a more or less professionalized form (Hofer, 2014), still trying to express authenticity (Stardust, 2019). Users involved in either non-commercial or commercial digital recording and sharing of their sexual interactions are vulnerable for consent violations in the form of wide circulation of the material against their will (so-called “revenge porn”; Eaton et al., 2020) or consent violations in the form of pressured or unwanted activities in front of the camera (Boyle, 2011).

### Causes and Consequences

What causes people to get involved in sexual interaction *through* digital technology, i.e., to search online for offline sexual partners and to consume or produce pornography during face-to-face sexual encounters? Research points to bio-psycho-social push factors meaning that particularly younger, non-heterosexual, male individuals with certain personality characteristics (e.g., sensation seeking) are more likely to get involved (Aretz et al., 2017; Wu and Ward, 2018; Bonilla-Zorita et al., 2020). Also, economic and technological pull factors that enable the respective behaviors need to be taken into consideration (e.g., convenient location-based search for potential partners through dating apps or easy access to smartphone camera and streaming apps during sexual encounters; Choi et al., 2017).

Regarding consequences of sexual interaction *through* digital technology, previous research has shown both negative and positive outcomes on general and sexual health. Involvement in sexual interaction through digital technologies could reduce sexual isolation, improve sexual satisfaction and provide validation of one’s own sexual identity (Hobbs et al., 2017), however, at the same time it is related to risks of sexual harassment or infidelity, addiction-like usage patterns and increased consumer mentality toward sexual partners (Couch et al., 2012; Choi et al., 2017). Consent turned out to be a key mediator as non-consensual interactions were linked to negative (Eaton et al., 2020) and consensual interactions to positive outcomes (Lemke and Merz, 2018). Consent is relevant in that regard not only for non-commercial, but also for commercial interactions: While the possibility to advertise own sexual services online has been economically beneficial for many sex workers (Mergenthaler and Yasseri, 2021), both private and commercial users are exposed to the risks of their intimate data being misused (Beckham and Prohaska, 2012; Greene-Colozzi et al., 2020).

## Sexual Interaction *VIA* Digital Technologies

Sexual interaction *via* digital technologies is a SIDC type that focuses on technology-mediated interpersonal sexual encounters.

### Three Subtypes

Three subtypes can be differentiated for sexual interaction *via* digital technology (see Table 5).

**TABLE 5:** Sexual interactions VIA digital technologies: subtypes.

**Subtype**	**Activity**	**Consent**	**Commerce**	**Example studies**
(1) Technology-mediated sexual interactions *via* digital media communication	Digital technologies are used to create a mediated text-, audio-, image-, photo- or video-based sexual interaction.	+	–	Beyens and Eggermont (2014) Carvalheira and Gomes (2003) Gregory (2018) Mileham (2007)
		–	–	Henry et al. (2018) Mandau (2020) Mckinlay and Lavis (2020) Naezer and van Oosterhout (2021)
		+	+	Bleakley (2014) Jones (2016) Selmi (2014) Weiss (2018)
		–	+	Açar (2017) Jones (2016)
(2) Technology-mediated sexual interactions *via* immersive virtual environments	Digital technologies are used to create a mediated avatar-based sexual interaction in virtual worlds.	+	–	Craft (2012) Gilbert et al. (2011) Mello et al. (2021) Wardle (2018)
		–	–	Behm-Morawitz and Schipper (2016) Reeves (2018) Strikwerda (2015)
		+	+	Lynch (2010) Martin (2014) Procter (2015) Smith (2009)
		–	+	/
(3) Technology-mediated sexual interactions *via* remote-controlled sex toys	Digital technologies are used to create a mediated sex toy-facilitated sexual interaction.	+	–	Flore and Pienaar (2020) Gomes and Wu (2018) Liberati (2017)
		–	–	Sparrow and Karas (2020) Sundén (2020) Wynn et al. (2017)
		+	+	Martins (2019)
		–	+	/

*Consent: “ + ” – consensual sexual interactions, “–“ – non-consensual sexual interactions. Commerce: “ + ” – commercial sexual interactions, “–“ – non-commercial sexual interactions.*

#### Technology-Mediated Sexual Interactions *via* Digital Media Communication

The first subtype addresses people experiencing technology-mediated sexual interactions *via* digital media communication (see Table 5). The technologically mediated sexual interaction can be based on digital text communication (often called “cybersex”; Carvalheira and Gomes, 2003), digital audio communication (often called “telephone sex”; Selmi, 2014), digital image communication (often called “sexting”; Döring, 2014) and/or digital video communication (often called “webcam sex” or “camming”; Henry and Farvid, 2017). According to the main mediators consent and commerce, the literature characterizes consensual non-commercial technology-mediated sexual encounters as creative and convenient forms of sexual intimacy for both singles and people in committed relationships (Beyens and Eggermont, 2014). At the same time, technology-mediated sexual interactions are described as risky, particularly because of boundary violations and non-consensual behaviors such as dissemination or publication of privately shared intimate messages against the will of the participant (e.g., so-called “revenge porn” or “image-based abuse”), non-consensual moves during the interaction (e.g., unsolicited sending of sexually explicit images such as “dick pics”) and technology-mediated sexual abuse of children (e.g., adults manipulating children into sending semi-nude pictures; Henry et al., 2018; Mandau, 2020; Mckinlay and Lavis, 2020; Naezer and van Oosterhout, 2021). In the context of commercial sex, the literature generally portrays technology-mediated sex work as a safer form of sexual labor that allows services providers even to become entrepreneurs (Podlas, 2000; Bleakley, 2014; Selmi, 2014; Jones, 2016; Weiss, 2018). At the same time, digital commercial sex services in the form of technology-mediated live interactions are associated with specific risks such as harassment or child prostitution (Jones, 2016; Açar, 2017; see Table 5).

#### Technology-Mediated Sexual Interactions *via* Immersive Virtual Environments

The second subtype concerns people experiencing technology-mediated sexual interactions *via* immersive virtual environments (see Table 5). A typical example of an immersive virtual environment is a virtual world such as Second Life. Virtual worlds are computer-simulated representations of fictional worlds, where users synchronously interact with each other via virtual representations of themselves called “avatars” (Bell, 2008). Among these interpersonal interactions are also sexual interactions often labeled as “avatar sex” (Wardle, 2018). People involved in avatar sex with each other can have very diverse relationship backgrounds ranging from being strangers to each other to being in a committed relationship (Craft, 2012). Avatar sex is described as somewhat more disinhibited, diverse and adventurous than participants’ real-life sex (Smith, 2009; Gilbert et al., 2011). Interestingly, experiments have shown that virtual touch of the avatar body one is wearing is related to erotic experiences (Mello et al., 2021).

Non-consensual practices, such as virtual rape or sexual interactions with avatars that look like children, are also known and have been met with vivid discussions in the research community regarding their ethical and legal implications (Strikwerda, 2015; Reeves, 2018). New emerging technologies, such as augmented reality (AR), become more and more widespread. While they are already starting to receive recognition in the context of sexual health education (Baran et al., 2020), the potential of AR systems for sexual interaction is still widely unknown (Lombard and Jones, 2013). Evidence about consensual commercial sex in immersive virtual environments focuses on Second Life and points to the fact that some people enjoy playing an avatar escort for both money and sexual satisfaction (Smith, 2009; Lynch, 2010; Martin, 2014; Procter, 2015).

#### Technology-Mediated Sexual Interactions *via* Remote-Controlled Sex Toys

The third subtype focuses on people experiencing technology-mediated sexual interactions *via* remote-controlled sex toys (see Table 5). Such smart sex toys that are controlled by a smartphone app via Bluetooth or Internet have become popular in recent years. Sex toy-facilitated sexual interaction over distance, which has been discussed for decades under the label “teledildonics” (Rheingold, 1990), is now available to end users (Flore and Pienaar, 2020). Integrating haptic interfaces in technology mediated sexual interactions provides additional sensual and erotic experiences (Liberati, 2017) and particularly supports people who lack physical touch such as couples in long-distance relationships or people with disabilities (Gomes and Wu, 2018). However, the design of haptic interfaces and the idea of “teledildonics” might also limit sexual expression by pushing a phallocentric or penetration-focused sexual script (Flore and Pienaar, 2020). Non-consensual uses of remote-controlled sex toys have already been identified such as sex toy producers illegally recording their customers’ sexual interactions via the toys (Sundén, 2020) or people deceiving their sex partners about their true identity to initiate sexual interactions via remote-controlled sex toys without their counterpart’s informed consent which turns the interaction to rape (Sparrow and Karas, 2020). Both privacy breaches and sexual assault are discussed as serious threads of smart sex toys (Wynn et al., 2017). Remote-controlled sex toys are also integrated in commercial sex, for example in commercial webcam live sex shows where the client pays to control the sex toys handled by the sex worker (Martins, 2019). The risks of consent violations described for non-commercial sexual interactions are applicable to commercial sex.

### Causes and Consequences

What causes people to get involved in sexual interaction *via* digital technology, i.e., to sexually interact with other people via digital media communication, remote-controlled sex toys or in immersive virtual environments? In regards to bio-psycho-social push factors, research indicates that younger, non-heterosexual, male individuals with specific personality characteristics (e.g., neuroticism, low levels of agreeableness) are more likely to get involved (Delevi and Weisskirch, 2013; Gordon-Messer et al., 2013; Courtice and Shaughnessy, 2017). Among economic and technological pull factors the emergence of affordable smart devices with aesthetically attractive design and the rapid growth of the Internet of Things (IoT) need to be considered (Flore and Pienaar, 2020).

Concerning consequences of sexual interaction *via* digital technology, previous research has reported both positive (e.g., opportunities for sexual self-exploration, pleasure, and sexual identity validation; Döring, 2014) and negative (e.g., increased involvement in sexual risk behaviors by adolescents; van Ouytsel et al., 2015) effects for general and sexual health of participants. Ñonsent plays a crucial role and is closely connected to technological factors, as non-consensual sexual interactions are often linked with data misuse and loss of control over one’s own sexual content resulting in humiliation, cyberbullying or harassment (Kopecký, 2015; Mckinlay and Lavis, 2020). In terms of the second mediator commerce, the literature suggests that providing technology-mediated live sexual services in a digital space creates a safer work environment compared to traditional offline settings and thus enhances the opportunity not only for the client, but also for the sex worker to experience sexual pleasure and satisfaction (Jones, 2016).

## Sexual Interaction *With* Digital Technologies

Sexual interaction *with* digital technologies is a SIDC type that focuses on sexual interactions where a digital artifact plays the role of the sex partner.

### Four Subtypes

With regard to sexual interaction *with* digital technologies four subtypes can be identified (see Table 6).

**TABLE 6:** Sexual interactions WITH digital technologies: subtypes.

**Subtype**	**Activity**	**Consent**	**Commerce**	**Example studies**
(1) Sexual interaction *with* media personas in traditional digital pornography	Digital technologies are used to enable sexual interaction between a person and media personas represented in text-, audio-, image- or video-based 2-dimensional pornographic content.	+	–	Ashton et al. (2019) Baudinette (2017) Döring (2021) Floegel (2020) Glascock and LaRose (1993) Gorissen (2020) Hester et al. (2015) Saunders (2019) Vaillancourt-Morel et al. (2017) Vučurović (2016) Zhang (2016)
		–	–	Eggestein and Knapp (2014) Harris (2019) Henshaw et al. (2017) Kikerpill (2020) McLelland (2010) Xu and Yang (2013)
		+	+	See section “Producing Digital Media Content during Face-to-Face Sexual Interactions”
		–	+	See section “Producing Digital Media Content during Face-to-Face Sexual Interactions”
(2) Sexual interaction *with* media personas in virtual reality pornography	Digital technologies are used to enable sexual interaction between a person and media personas represented in virtual reality pornography.	+	–	Dekker et al. (2021) Elsey et al. (2019) Simon and Greitemeyer (2019)
		–	–	/
		+	+	/
		–	+	/
(3) Sexual interaction *with* software sexbots	Digital technologies are used to enable sexual interaction between a person and software sexbots.	+	–	Banks and van Ouytsel (2020) Liu (2021)
		–	–	Laorden et al. (2015) Curry and Rieser (2018)
		+	+	/
		–	+	/
(4) Sexual interaction *with* hardware sex robots	Digital technologies are used to enable sexual interaction between a person and a physical AI-enabled sex robot.	+	–	Appel et al. (2019) Döring et al. (2020) González-González et al. (2020) Jecker (2021) Nordmo et al. (2020) Oleksy and Wnuk (2021) Szczuka and Krämer (2017a) Szczuka and Krämer (2017b)
		–	–	Brown and Shelling (2019) Danaher (2017) Frank and Nyholm (2017) Maras and Shapiro (2017) Richardson (2016) Zara et al. (2021)
		+	+	Yeoman and Mars (2012)
		–	+	Richardson (2016)

*Consent: “ + ” – consensual sexual interactions, “–“ – non-consensual sexual interactions. Commerce: “ + ” – commercial sexual interactions, “–“ – non-commercial sexual interactions.*

#### Sexual Interaction *With* Media Personas in Traditional Digital Pornography

The first subtype addresses people engaging in sexual interaction *with* media personas represented in traditional digital pornography (see Table 6). Traditional digital pornography comes in a variety of media forms. The literature differentiates between text-based (e.g., erotic fan fiction; Floegel, 2020; Döring, 2021), audio-based (e.g., adult phone line recordings; Glascock and LaRose, 1993), image-based and computer-generated (e.g., erotic manga and anime; Vučurović, 2016; Baudinette, 2017; erotic gifs; Hester et al., 2015; GGI porn; Saunders, 2019) and/or video-based digital pornographic content (e.g., cyberporn; Ashton et al., 2019). Research on traditional digital pornography mostly focuses on video-based or 2-dimensional (2D) audiovisual digital pornography. All these traditional forms of pornography allow the user to engage in parasocial interactions and relationships with the media personas (e.g., with the porn performers in video-based porn). Contemporary porn performers foster the parasocial interactions and relationships with their viewers and fans by presenting themselves not only in pornographic videos but also on social media platforms such as Twitter to appear more approachable and real (Gorissen, 2020).

Current studies show that people of different genders and sexual orientations voluntarily use traditional digital pornography that they can access discreetly and often cost-free on digital platforms (Vaillancourt-Morel et al., 2017). Issues of consent come up when adults and minors experience unwanted exposure to digital pornography (e.g., confrontation with pornographic pop-up adverts on the Internet or with forwarded porn images on social media) and when people are using sexually explicit content that depicts real life child sexual abuse (e.g., so-called “child pornography”; Eggestein and Knapp, 2014; Henshaw et al., 2017). Widely debated in the context of consent are also fictional pornographic descriptions or depictions of rape scenes, incest or sex with minors (McLelland, 2010). Furthermore, current technology allows to produce fake pornographic images and videos from everyone (so-called “deep fake”) against their will which poses a threat to their reputation (Harris, 2019; Kikerpill, 2020).

Most research on pornography has focused on its users. Research on professional porn performers and their involvement in commercial porn production is scarce. From the perspective of the porn actor participation in commercial porn production means that their face-to-face sexual interactions are recorded. Hence this activity would fall in the first type of SIDC: sexual interaction *through* digital technology (subtype: Producing digital media content during face-to-face sexual interactions; see section “Producing Digital Media Content during Face-to-Face Sexual Interactions”).

#### Sexual Interaction *With* Media Personas in Virtual Reality Pornography

The second subtype deals with 3-dimensional (3D) audiovisual digital pornography (see Table 6). It is commonly labeled in the literature as “VR porn” (Wood et al., 2017). Virtual reality (VR) pornography has been called a game-changer for the whole porn industry due to the vivid experiences it provides (Elsey et al., 2019). VR pornography is used not in front of a display but with a VR headset that allows to immerse in the VR world. By turning the head, the user can look around in the virtual scene. Some VR porn videos are filmed from the perspective of an external observer (voyeuristic perspective), others form the point of view (POV) of the user so that the illusion is created that the user has sex with the porn actor. Experimental studies have demonstrated that immersive VR porn provides a more intense and exciting experience than the use of traditional digital pornography on a desktop screen, particularly when the scene is filmed from the POV of the user (Elsey et al., 2019; Simon and Greitemeyer, 2019; Dekker et al., 2021). Particularly, the parasocial interaction seems to be stronger: In the VR condition male users felt more desired, more flirted with and more looked in the eyes by the porn performer (Dekker et al., 2021).

So far, the research has focused on voluntary use. Issues of consent and commerce have not yet been investigated in the context of 3D or virtual reality pornography.

#### Sexual Interaction *With* Software Sexbots

The third subtype focuses on sexual interactions with software sexbots (e.g., AI-enabled chatbots; Banks and van Ouytsel, 2020; AI-enabled holograms; Liu, 2021). Those software artifacts are not tangible but invite the user to engage in social, romantic and sexual interactions and relationships. The 2013 science fiction US-movie ‘‘Her’’ has illustrated how a man can fall in love with an intelligent chatbot that is only present via its voice. However, available sex chatbots are mostly not yet audio-based but purely text-based^1^.

Issues of consent arise when sexbots appear childlike or when users engage in aggressive and abusive interactions with virtual assistants (Laorden et al., 2015; Curry and Rieser, 2018). So far, the use of software sexbots in commercial sex has not been explored.

#### Sexual Interaction *With* Hardware Sex Robots

The fourth subtype addresses sexual interactions with hardware sex robots (see Table 6). Hardware sex robots differ from software sexbots in terms of their materiality. They are often described as AI-enhanced sex dolls (Döring et al., 2020; González-González et al., 2020). Hardware sex robots stand out from all other technologies discussed in this paper so far because of their high price of several thousand US Dollars. Even though the first sex robot models are on the market^2^, the community of pioneer users of sex robots seems to be fairly small and not very visible in the public (Döring et al., 2020).

That is why empirical research on sex robots so far often draws on sex dolls and sex doll owners as a proxy as this user group is larger and also more publicly visible and accessible via their sex doll owner online communities (Döring and Pöschl, 2018). Research on sex doll owners has revealed a diversity of use patterns that include sexual interaction (e.g., sexual intercourse with the doll) but also social interaction (e.g., dining and watching TV with the doll) as well as physical care work (e.g., washing, powdering, and dressing the doll). Another research strand explores attitudes toward sex robots, intentions to use and to buy a sex robot with the help of surveys and vignette experiments where the robot is described to the participants or pictures of the robot are shown (Szczuka and Krämer, 2017a,b; Appel et al., 2019; Nordmo et al., 2020; Oleksy and Wnuk, 2021). Even though first theoretical models of the psychological mechanisms of sex robot use have been presented (Szczuka et al., 2019), so far, no empirical data are available on sexual and social interactions with actual sex robots or about long-term sex robot users.

The emerging technology of sex robots has elicited a lot of ethical and legal concerns around issues of consent. Main objections are that male users assault and rape female sex robots as well as child-like robots and hence normalize and train sexually abusive and violent behaviors (Richardson, 2016; Danaher, 2017; Frank and Nyholm, 2017). Child-like sex dolls and sex robots are considered particularly dangerous, and their production and possession is already criminalized in some countries (Maras and Shapiro, 2017; Brown and Shelling, 2019). The idea that female or child-like sex dolls and sex robots could successfully be used in the therapy of sex offenders is met with skepticism by ethicists, therapists, and sex offenders (Zara et al., 2021).

Commercial use of sex robots is already being observed in the sense that selected brothels world-wide offer users the option to rent a sex doll/robot or to book a sex worker together with a sex doll/robot (Döring et al., 2020). Some authors speculate that the use of sex robots in commercial sex could be beneficial. When sex robots substitute female prostitutes (e.g., in the red light district of Amsterdam) related risks of sexual violence, sex trafficking and STI transmission become obsolete (Yeoman and Mars, 2012). Other authors, however, argue that using a female sex robots as prostitutes and sex slaves is already an act of symbolic violence against all women and will foster more sexual violence against women and children (Richardson, 2016).

### Causes and Consequences

What causes people to get involved in sexual interaction *with* digital technology, i.e., to sexually interact with traditional or virtual reality pornography, with software sexbots or hardware sex robots? Taking into account bio-psycho-social push factors, research showed that men are more likely than women to interact with digital pornography and to engage in sexual interactions with sex robots (Döring et al., 2020). Such traits as sensation seeking, high sexual motivation, lower self-esteem and an overall positive attitude toward new technologies were also associated with the more eager engagement in sexual interaction with digital technologies (Laier and Brand, 2014; Stark et al., 2018; Szczuka and Krämer, 2019). Socio-cultural factors in terms of positive attitudes toward intimate interactions with technology play an important role (Aoki and Kimura, 2021). In terms of economic and technological pull factors, the wide spread of sexually explicit content on the web (Cooper et al., 2004) and the attractive human-like design, functionality and wide media representation of sex robots (Döring and Poeschl, 2019) are important motivations for their use.

Regarding consequences of sexual interaction with digital technology, previous research has pointed both to risks and opportunities for general and sexual health. It appears that some characteristics of the technology are ambivalent and might create positive and negative effects. For example, sexual interaction with novel digital technologies such as seemingly interactive media persona in immersive virtual pornography or tangible intelligent sex robots can enhance sexual arousal and satisfaction (Dekker et al., 2021) while, at the same time, this technology can increase risks of overuse and addiction (Laier and Brand, 2017; Brand et al., 2019, 2020). Customization of digital technologies can also be ambivalent. On the one hand, catering to the user’s sexual preferences in terms of a sex robot appearance, personality and behaviors might enhance their well-being and arousal, while on the other hand, customized pornography and sex robots might feed into objectification, exaggerated beauty standards and unrealistic expectations that, ultimately, endanger sexual satisfaction (Döring et al., 2020). Interaction with technological artifacts frees users from social rules of interpersonal interactions, which can improve well-being and satisfaction. At the same time, sexual interactions with human-like (mostly woman-like) artifacts violating usual social norms of sexual consent, are assumed to be linked with very negative psychological and social outcomes (Richardson, 2016). Furthermore, the design potentials of innovative digital technologies promise to fulfill sexual fantasies and, hence, foster well-being, while they also bring about risks of privacy violations (Kikerpill, 2020; Ratner, 2021).

## Discussion

The present paper introduces a new conceptual model of SIDC that is arousal-oriented and technology-inclusive. Considering that digital technologies can not only mediate interpersonal sexual interactions, but also act as an equal partner in it, the model differentiates between sexual interaction *through*, *via* and *with* digital technologies, depending on the role the technology plays in the interaction. Consent and commerce are identified as relevant mediators. Both causes and consequences of SIDC are described as multidimensional including bio-psycho-social as well as economic and technological factors.

### Reflections on the Model and the State of Research

The core and most innovative aspect of our model is the distinction between the three types of sexual interactions: Those that occur *through* digital media, *via* digital media and *with* digital media. By introducing this systematization, we highlight that sexual interaction in the context of digital media is diverse and that digital media can take very different roles in sexual interactions. As the overview of current research indicates, first evidence that these different roles lead to different effects can be identified. Still, future research needs to scrutinize more systematically which interaction type entails which risks and benefits. So far, the summary of recent results clearly indicates that sexual interaction in digital contexts is not only related to risks but can also be accompanied by beneficial outcomes – contradicting early assumptions and publicly discussed fears (Döring, 2009). Future research, however, will need to more systematically assess the magnitude of risks and benefits and the specific boundary conditions which lead to each.

In this line, another important addition to current research and theorizing is that we plead to not only include bio-psycho-social causes and consequences as has been argued before (Shaeer et al., 2017; Leavitt et al., 2021) but to also consider economic and, specifically, technological causes and consequences. A further addition to current theorizing is the inclusion of the mediators consent and commerce. Here, the overview about first research that either focusses on (non)consensual and/or (non)commercial sexual interactions or even directly addresses their differences indicates that these mediators indeed affect the relationship between the digitalized sexual interaction and the consequences. Future research needs to scrutinize the boundary conditions. Therefore, altogether this contribution is meant to support future research by identifying variables that have so far been overlooked and that have not been sufficiently systematized. Still, the strength of the paper lies in its summary of relevant phenomena, variables and empirical findings. The model we present, however, is not yet a theoretical model. Future research and theorizing must clarify how the variables and conditions which we identified interact and what exact mechanisms lead to risks or benefits.

### Limitations

Despite its universal approach, the presented conceptual analysis of sexual interaction in digital contexts has its limitations. The analysis has been conducted by an interdisciplinary research team with experts from psychology, medicine, and communication science. It was validated by discussions with further colleagues from these three fields and related social sciences (e.g., sociology). While all experts involved are familiar with relevant research in engineering (e.g., smartphone apps, virtual reality systems, robotics), genuine engineers or computer scientists did not participate. The conceptual analysis methodology involved searching for and analyzing a large body of interdisciplinary literature that is presented both in text and tables. However, it has not been the goal of this paper to provide a systematic literature review. As our literature search was limited to English-language academic sources and our research group is based in Germany, we need to admit that our perspective might have a bias toward the Western world and the Global North.

There is broad consensus that SIDC can have negative (e.g., sexual addiction, sexual violence, relationship breakup) as well as positive (e.g., sexual self-validation, sexual satisfaction, relationship building) consequences. Also, it is obvious that SIDC consequences on different dimensions influence each other: For example, using digital media to search for face-to-face sexual interactions can lead to infidelity among partnered and married participants. Infidelity as a social consequence of online dating can bring about relationship crisis and breakup, emotional stress and increased cortisol level as psychological and biological consequences, and even economic consequences such as loss of property and assets. All of this is covered by our SIDC model. However, we did not explicitly differentiate short-, medium-, and long-term consequences. In the case of a SIDC-induced relationship breakup or divorce, short- and medium-term consequences can be experienced as very negative, while long-term evaluations can be positive, for example in light of a happy and sexually fulfilling new relationship. This example illustrates that classifying SIDC consequences as positive or negative, as opportunities or challenges for sexual and overall health is often not straightforward. People involved in SIDC, external observers, and clinical experts alike can be ambivalent or unsure about the valence of consequences and might change their evaluation over time. The value-laden and even philosophical issue of evaluating complex SIDC consequences from a lifespan-perspective is beyond the scope of this analysis.

### Conclusion and Outlook

The aim of the present paper was to identify relevant variables that so far have been overlooked or under-researched. By differentiating the three types of sexual interaction *through, via* and *with* technology and discussing them together with bio-psycho-social-economic-technological causes and consequences and with mediators such as consent and commerce, we lay the ground for future studies in this broad and emerging field.

It would, for instance, be helpful to design comparative studies, that contrast different (sub)types of SIDC, e.g., interactions with media personas in traditional 2D versus 3D digital pornography or interactions with software versus hardware sexbots. Those comparisons can be helpful to better understand experiences of social presence and para-sociality during sexual interactions with technology and their links to sexual arousal and satisfaction. Based on the current state of research it is expected, for example, that interaction with media personas in 3D porn are experienced as more intense in comparison to interactions with media personas in 2D porn. Furthermore, interaction with a hardware sexbot should provide more intense experiences than interactions with a software sexbot.

Also, acknowledging consent and commerce as meaningful mediators of SIDC, leads to questions of precise measurement: Using instruments that clearly distinguish between consensual and non-consensual as well as between commercial and non-commercial SIDC is an important first step. However, research points to the need to further differentiate between different qualities or degrees of consent and commerce (e.g., consensual but still unwanted sexting or unpaid but still incentivized sex). Here more research is needed to explore, for example, the different causes, characteristics and consequences of online sex dating that (a) involves no commercial benefit, (b) provides incentives such as invitations to dinners and events or other gifts, (c) includes the direct exchange of money. So far, no validated measures have been developed to collect data on different types of commercial sex in digital contexts such as regular sex dating apps and so-called “sugaring” apps.

Furthermore, we encourage studies that cover causes and consequences in a multi-dimensional way and measure both negative and positive consequences for sexual and overall health in a balanced way. For example, engaging in commercial sex in digital context can be both beneficial and detrimental to general and sexual health (e.g., relief from financial stress and related health issues but at the same time increased vulnerability to STI transmission and stigmatization and, hence, increase in related health issues).

A multi-dimensional analysis is also recommended, according to our SIDC model, when it comes to analyzing the causes or predictors of certain effects of sexual interaction in digital contexts. For example, factors contributing to successful online sex dating can be biological, psychological, social, technological, and economic.

The proposed new SIDC model is meant to help identify research gaps (see, e.g., the empty cells in Tables 4–6) and to provide a systematic framework that assists in incorporating the most relevant variables in future research. To put the model to research practice we suggest to design studies that incorporate those variables that have been neglected. For example, Tables 4–6 reveal that we don’t know much about the incorporation of technologies in commercial sex (e.g., use of pornography, sex dolls or sex robots during interactions with a sex worker).

Last not least we need to point out that much work still needs to be done: The systematization and theoretical framework we present here need to be transformed to a theoretical model which specifies causal mechanisms and processes. Moreover, a long-term or lifespan-perspective when investigating SIDC needs to be incorporated. Ever new and emerging technologies will make sure that the prospective model needs to be constantly checked for potential amendments (e.g., incorporation of new subtypes of SIDC) based on new socio-technical developments.

## Data Availability Statement

The original contributions presented in the study are included in the article/supplementary material, further inquiries can be directed to the corresponding author.

## Author Contributions

ND, NK, MB, TK, and GV contributed to planning the manuscript, doing the conceptual analysis, developing the conceptual model, and revising the manuscript. ND and VM took the lead in writing and editing the manuscript. All authors contributed to the article and approved the submitted version.

## Conflict of Interest

The authors declare that the research was conducted in the absence of any commercial or financial relationships that could be construed as a potential conflict of interest.

## Publisher’s Note

All claims expressed in this article are solely those of the authors and do not necessarily represent those of their affiliated organizations, or those of the publisher, the editors and the reviewers. Any product that may be evaluated in this article, or claim that may be made by its manufacturer, is not guaranteed or endorsed by the publisher.

## Footnotes

1 e.g., collection of adult sex bots, https://www.personalityforge.com/adult-chatbots.php

2 e.g., the AI driven robotic doll “Harmony-X” from the US company Real Doll: https://www.realdoll.com/product/harmony-x/
