# Corrigendum: Sexual Interaction in Digital Contexts and Its Implications for Sexual Health: A Conceptual Analysis

**DOI:** 10.3389/fpsyg.2022.847814

**Published:** 2022-02-09

**Authors:** Nicola Döring, Nicole Krämer, Veronika Mikhailova, Matthias Brand, Tillmann H. C. Krüger, Gerhard Vowe

**Affiliations:** ^1^Media Psychology and Media Design, Institute of Media and Communication Science, Department of Economic Sciences and Media, Technische Universität Ilmenau, Ilmenau, Germany; ^2^Social Psychology: Media and Communication, Department of Computer Science and Applied Cognitive Science, Faculty of Engineering, University of Duisburg-Essen, Duisburg, Germany; ^3^General Psychology: Cognition and Center for Behavioral Addiction Research (CeBAR), Department of Computer Science and Applied Cognitive Science, Faculty of Engineering, University of Duisburg-Essen, Duisburg, Germany; ^4^Department of Psychiatry, Social Psychiatry and Psychotherapy, Section of Clinical Psychology and Sexual Medicine, Hannover Medical School, Hannover, Germany; ^5^Communication and Media Studies, Center for Advanced Internet Studies (CAIS), Bochum, Germany

**Keywords:** internet sexuality, cybersex, online sexual activities (OSA), sexting, pornography, sex robots, sexual consent, commercial sex

There was an error in the correspondence details as published. Nicole Krämer was not included as a corresponding author in the published article. The corrected correspondence details appear below.

Correspondence: Nicola Döring, nicola.doering@tu-ilmenau.de; Nicole Krämer, nicole.kraemer@uni-due.de.

The authors apologize for this error and state that this does not change the scientific conclusions of the article in any way. The original article has been updated.

## Publisher's Note

All claims expressed in this article are solely those of the authors and do not necessarily represent those of their affiliated organizations, or those of the publisher, the editors and the reviewers. Any product that may be evaluated in this article, or claim that may be made by its manufacturer, is not guaranteed or endorsed by the publisher.

